# Valuable Predictors for Non-measurability of Fractional Flow Reserve Derived From Coronary Computed Tomography Angiography

**DOI:** 10.7759/cureus.59227

**Published:** 2024-04-28

**Authors:** Hideaki Nonaka, Kazuyuki Yahagi, Kota Komiyama, Yuki Gonda, Yu Horiuchi, Masahiko Asami, Hitomi Yuzawa, Jun Tanaka, Jiro Aoki, Kengo Tanabe

**Affiliations:** 1 Division of Cardiology, Mitsui Memorial Hospital, Tokyo, JPN

**Keywords:** fractional flow reserve derived from coronary computed tomography angiography, non-measurability, heart rate, ffrct, coronary computed tomography angiography, coronary calcium score, coronary artery disease

## Abstract

Background

The fractional flow reserve (FFR) derived from coronary computed tomography (CT) angiography (FFRCT) is a variable tool for coronary disease diagnosis that non-invasively provides the value of FFR. It can add physiological information to coronary CT angiography (CCTA) and reduce unnecessary invasive coronary angiography (CAG). However, it cannot be analyzed in some cases, which is also called “non-measurability.” While FFRCT has become globally widespread, the current data on non-measurability are lacking. This study aimed to determine the rate of non-measurability and identify predictors thereof in routine clinical settings to explore potential approaches to reduce the non-measurability rate.

Methods and results

This retrospective observational single-center study included consecutive patients who underwent FFRCTanalysis in Japan. The mean age of the overall population was 71.3 ± 10.6, and an FFRCTof ≤0.8 was seen in 47.6% of patients with a measurable FFRCT. Of the 307 enrolled patients, FFRCT analysis was not feasible in 21 cases (6.8%). Heart rate (HR) at a CT scan and coronary calcium scores (CCS) were significantly higher in patients with non-measurability than those in patients whose FFRCT was appropriately analyzed (HR: 69.6±8.9 bpm vs. 61.0±11.1 bpm; p < 0.01; CCS; 931.2 (290.8, 1451.3) vs. 322.9 (100.7, 850.0); p < 0.01). Multiple logistic regression showed that HR was an independent predictor for non-measurability (odds ratio: 1.05; 95% confidential interval: 1.02, 1.09; p < 0.01)). Based on the receiver operating characteristic curve analysis, the optimal cut-off value of HR and CCS was 63 bpm (specificity: 67.1%; sensitivity: 76.2%) and 729.2 (specificity: 71.3%; sensitivity: 66.7%). In addition, the combination of two features (HR > 63 bpm and CCS > 729.2) showed a high negative predictive value (99.3%) for FFRCT non-measurability.

Conclusions

In this study, the rate of FFRCTnon-measurability was 6.8%. Higher HR at a CT scan and CCS were significantly associated with non-measurability, and in cases with both HR and CCS below a specified threshold, the likelihood of ruling out non-measurability could be significantly high. Our findings suggest that reducing the HR to ideally under 63 bpm at the time of the CT scan significantly ensures feasibility. Further study on large-scale cohorts is warranted.

## Introduction

Coronary artery disease remains a common problem in modern clinical practice [1] and is a leading cause of mortality [2,3]. Invasive fractional flow reserve (FFR), providing us with the physiological severity of coronary stenosis, has conventionally been recommended to assess the need for therapeutic intervention [2,4]. Recently, a non-invasive method to calculate FFR via coronary computed tomographic angiography (CCTA) image has been developed, called "FFRCT" [3]. It employs computational fluid dynamics and image-based modeling, permitting the determination of rest and hyperemic coronary flow and pressure from CCTA scans, without additional imaging, modification of acquisition protocols, or administration of medications. In clinical settings, this advanced technique enables us to gain FFR value non-invasively along with CCTA and to reduce unnecessary invasive coronary angiography, potentially decreasing the burden on patients and healthcare costs [5-8]. The previous multicentre studies have demonstrated its high diagnostic accuracy and discrimination with invasive FFR as the reference standard (area under the curve: 0.9) [6] and its high predictive value for fewer adverse cardiac events [7]. Although the utility of FFRCT has been established and is becoming indispensable in current clinical settings, the analysis can be rejected from the hub site of analysis at times, which is also called “non-measurability”, and its rate was previously reported as 8.4% in 2019 [9]. Although the analysis software has significantly advanced thereafter, the updated data on the non-measurability have not been reported. In this study, we aimed to determine the current rate of non-measurability and identify predictors thereof in routine clinical settings to explore potential approaches to reduce the non-measurability rate.

## Materials and methods

Study design and population

**Figure 1:**
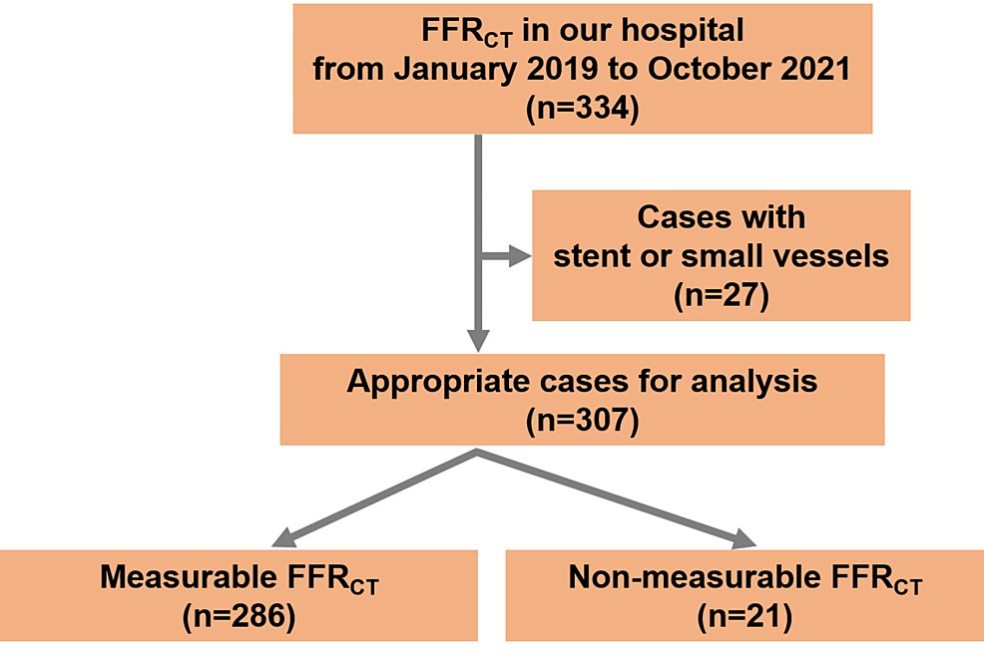
The flowchart of the patient selection A flowchart demonstrates the selection process of the study population. FFRCT, fractional flow reserve derived from coronary computed tomography angiography

The study retrospectively analyzed 334 consecutive cases using FFRCT analysis at a Japanese institution from January 2019 to October 2021 (Figure 1), detailing patient selection criteria, imaging protocols, and analytic techniques. The decision to refer patients for FFRCT analysis was made by the physician interpreting the CCTA based on the abnormalities in electrocardiogram, echocardiography, or chest symptoms suspected of coronary artery stenosis. Among those patients, those with a previous history of revascularization (percutaneous coronary intervention with a stent or a coronary artery bypass graft; n=26) and small vessels (≤1.8 mm; n=1) were excluded, as in previous studies [9]. This is because revascularization history and small vessels are actually not recommended for FFR-CT analysis at HeartFlow Inc. (Redwood City, CA), which is the site where our FFR-CT analysis was conducted. The present study was conducted in accordance with the Declaration of Helsinki and approved by the local institutional review committee on human research (Mitsui Memorial Hospital Ethics Committee No. C26). The study details were explained to all participants when obtaining consent for the CCTA scan and FFRCT analysis, and informed consent for this study was obtained in an opt-out manner.

CCTA acquisition, reconstruction, and analysis

CCTA was conducted using single-source computed tomography (CT) scanners with 320 detector rows (Aqualion One, Canon Medical Systems, Tochigi, Japan) based on a previously described protocol [10]. Scanning parameters were as follows: collimation of 320 rows x 0.5 mm, rotation time of 350-400 ms, and tube voltage of 120 kV. The tube current (270-400 mA) was chosen based on the standard deviation of the noise level measured on the CT projection radiographs. Electrocardiogram-gated non-enhanced scans were obtained, followed by contrast-enhanced scans using Iopamiron 370 mg/mL injected at a rate of 4.0-5.0 mL/s. The scanner’s “arrhythmia rejection” software automatically removed irregular beats using the multi-segment acquisition technique. We semi-automatically determined the appropriate cardiac phase with minimal cardiac motion for the CT axial image reconstruction using the Phase-NAVI scanner software (Canon Medical Systems, Tochigi, Japan). All data acquired with 0.25-mm-thick slices were reconstructed using a standard kernel of FC04. Oral or intravenous beta-blockers, or both, were administered to achieve a heart rate (HR) of ≤60 beats/min [11], and sublingual nitroglycerin was administered to patients without any contraindications. The coronary artery calcium score (CCS) was calculated using a workstation (Ziostation2, Ziosoft, Tokyo, Japan) and expressed using the previously reported Agatston score [12].

FFRCT analysis

All FFRCT analyses were performed at HeartFlow Inc. (Redwood City, CA) as previously described [13]. Their software applies computational fluid dynamics and deep-learning image-based modeling to estimate rest and hyperaemic coronary flow and pressure from CCTA. Non-measurability in the FFRCT analysis was defined as an inability to perform quantitative analysis with FFRCT [14] and was determined by HeartFlow Inc.

Statistical analysis

Categorical variables were expressed as numbers and percentages (%), and continuous variables were expressed as mean±standard deviation (SD) or median and interquartile range (IQR) (25th to 75th percentile) according to their distribution. Assessment of normality for continuous data was performed using the Kolmogorov-Smirnov test. Categorical variables were analyzed using the chi-square or Fisher’s exact test, and differences in continuous variables were analyzed using a two-sided Student’s t-test or the Wilcoxon rank test. Binary logistic regression was used to assess the association between baseline covariates and non-measurability in FFRCT analysis (results were presented as odds ratio (OR) and 95% confidence interval (CI)). In order to predict the non-measurability, the optimal cut-off value with sensitivity and specificity was determined by the receiver operating characteristic (ROC) curve analysis. Variables with p<0.05 were considered to indicate a significant difference. All analyses were performed using the R software (version 4.1.2; R Foundation for Statistical Computing, Vienna, Austria). To control for type I error inflation due to multiple comparisons, we employed the Benjamini-Hochberg procedure. The false discovery rate was set at 5%.

Preprint disclosure

This article was previously posted to the Research Square preprint server (doi.org/10.21203/rs.3.rs-3211999/v1) on 2nd August 2023.

## Results

Patient characteristics

We analyzed 307 cases in Japan, and their main clinical characteristics are summarized in Table 1. The mean age was 71.3 ± 10.6 years old. The rate of males (67.8%), smokers (43.0%), dyslipidemia (58.6%), and hypertension (69.4) were relatively high in the overall population. Non-measurability occurred in 21 (6.8%) patients and was associated with a higher HR at CT scanning (p < 0.01), higher CCS (p < 0.01), and lower ejection fraction (p = 0.047) than those with their FFRCT measurable. The rates of beta-blockers, nitroglycerin use, and atrial fibrillation were comparable between the two groups. A FFRCT of ≤0.8 was seen in 47.6% of patients with a measurable FFRCT, and a FFRCT of ≤0.8 was seen in 23.1% of all vessels with a measurable FFRCT.

**Table 1:** Baseline characteristics FFRCT, fractional flow reserve derived from coronary computed tomography angiography; BMI, body mass index; MI, myocardial infarction; TG, triglyceride; HDL-C, high-density lipoprotein cholesterol; TC, total cholesterol; eGFR, estimated glomerular filtration rate; HbA1c, hemoglobin A1c, NT-proBNP, N-terminal prohormone of brain natriuretic peptide; AF, atrial fibrillation

	Overall (n=307)	Measurable FFRCT (n=286)	Non-measurable FFRCT (n=21)	P value
Age (y)	71.3 ± 10.6	71.0 ± 10.6	74.4 ± 10.8	0.163
Female (%)	99 (32.2)	92 (32.2)	7 (33.3)	1.000
BMI (kg/m^2^)	24.9± 15.2	25.1 ± 15.7	22.4 ± 0.3.7	0.426
Smoking history (%)	132 (43.0)	125 (43.7)	7 (33.3)	0.485
Dyslipidemia (%)	180 (58.6)	169 (59.1)	11 (52.4)	0.709
Diabetes mellitus (%)	83 (27.0)	76 (26.6)	7 (33.3)	0.675
Hypertension (%)	213 (69.4)	201 (70.3)	12 (57.1)	0.310
Hemodialysis (%)	16 (5.2)	15 (5.2)	1 (4.8)	1.000
Previous MI	5 (1.6)	5 (1.9)	0 (0)	1.000
TG (mg/dL)	140.1 ± 121.8	143.1 ± 124.5	100.1 ± 65.7	0.119
HDL-C (mg/dL)	59.3 ± 24.1	59.5 ± 24.4	56.3 ± 20.1	0.648
TC (mg/dL)	175.1 ± 70.0	175.2 ± 70.5	173.6 ± 65.5	0.917
eGFR (mL/min/1.73m^2^)	61.2 ± 20.2	61.3 ± 20.4	60.3 ± 18.2	0.879
HbA1c (%)	6.2 ± 3.2	6.3 ± 3.2	5.6 ± 2.6	0.311
NT-proBNP (pg/mL)	102.0 (35.5, 238.5)	97.0 (35.3, 241.0)	124.0 (47.0, 209.0)	0.836
Heart rate at scan (bpm)	61.6 ± 11.1	61.0 ± 11.1	69.6 ± 8.9	0.001
Total calcium score	356.5 (108.8, 899.8)	322.9 (100.7, 850.0)	931.2 (290.8, 1451.3)	0.008
Amount of contrast (mL)	43.2 ± 13.6	43.1 ± 13.8	44.9 ± 11.0	0.556
Ejection fraction (%)	63.7 ± 11.4	64.1 ± 10.9	60.0 ± 16.3	0.047
Use of β-blocker (%)	138 (45.0)	126 (44.1)	12 (57.1)	0.349
Use of nitroglycerin (%)	267 (87.0)	247 (86.4)	20 (95.2)	0.406
AF at scan (%)	26 (8.5)	24 (8.4)	2 (9.5)	1.000

Reason for non-measurability

**Figure 2:**
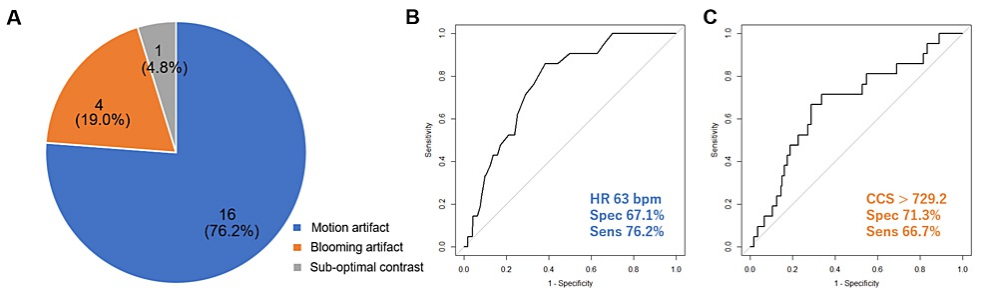
The reasons for FFRCT non-measurability (A) and the ROC curve for FFRCT non-measurability (B,C) The pie chart (A) shows reasons for FFRCT non-measurability. The major reasons were motion and blooming artifacts. The ROC curve (B) shows the predictive value of HR for non-measurability (cut-off value, 63 bpm; AUC: 0.77; sensitivity: 67.1%; specificity: 76.2%). The other ROC curve (C) shows the predictive value of CCS for non-measurability (cut-off value, 729.2; AUC: 0.67; sensitivity: 71.3%; specificity: 66.7%) FFRCT, fractional flow reserve derived from coronary computed tomography angiography; ROC, receiver-operating characteristic; HR, heart rate; AUC, area under the curve; CCS, coronary artery calcium score

According to the analysis by HeartFlow Inc., motion artifact (n=16; 76.2%) and blooming artifact (n=4; 19.0%) were major reasons for non-measurability (Figure 2A), followed by sub-optimal contrast (n=1; 4.8%). Data from all 21 cases with rejected FFRCT analysis are presented in Table 2. The right coronary artery (RCA) (n=16; 76.2%) was the most common non-measurable vessel.

**Table 2:** Presentations of each case with their FFRCT non-measurable FFRCT, fractional flow reserve derived from coronary computed tomography angiography; HR, heart rate; CCS, coronary artery calcium score; RCA, right coronary artery; LMT, left main trunk; LAD, left anterior descending artery; LCX, left circumflex artery

Age (y)	Sex	HR (bpm)	CCS	Non-measurable vessel	The reason for FFRCT non-measurability
75	M	57	48.7	ALL	Sub-optimal contrast
88	M	65	2192.0	RCA	Blooming artifact
75	F	64	931.2	RCA	Blooming artifact
73	F	63	1299.5	RCA	Blooming artifact
79	M	56	789.2	LMT	Blooming artifact
78	M	68	260.7	RCA	Motion artifact
52	M	80	1657.3	RCA	Motion artifact
69	F	66	1212.7	RCA	Motion artifact
86	M	80	1383.5	RCA	Motion artifact
64	F	72	7.2	RCA	Motion artifact
81	F	95	606.3	RCA	Motion artifact
59	M	76	149.2	RCA	Motion artifact
90	F	66	290.8	RCA	Motion artifact
83	M	63	743.5	RCA	Motion artifact
78	M	61	1845.1	RCA	Motion artifact
73	M	74	731.6	RCA	Motion artifact
74	M	74	34.5	ALL	Motion artifact
53	M	75	3451.4	ALL	Motion artifact
73	M	69	1154.3	LAD	Motion artifact
90	F	65	1451.3	LAD	Motion artifact
69	M	71	2847.4	LCX	Motion artifact

Factors associated with non-measurability

Univariate logistic regression analyses revealed that HR at CT examination (OR: 1.004; 95% CI: 1.001-1.007; p < 0.01) and CCS (OR: 1.00; 95% CI: 1.00-1.00; p = 0.03) were significant factors associated with non-measurability. Although we additionally analyzed other variables, including the amount of contrast, use of β-blocker or nitroglycerin, and AF, no significance was observed. Due to the small number of events, we conducted multivariate logistic regression analysis only including HR and CCS, and HR was the independent factor for FFRCT non-measurability (Table 3) (OR: 1.004; 95% CI: 1.001-1.006; p < 0.01). Based on the ROC curve analysis, the optimal cut-off for HR to predict non-measurability was 63 bpm, with a specificity and sensitivity of 67.1% and 76.2%, respectively (Figure 2B). The area under the ROC curve (AUC) was 0.77 (95% CI: 0.68-0.85). The CCS’s optimal cut-off to predict non-measurability was 729.2 with a specificity and sensitivity of 71.3% and 66.7%, respectively (Figure 2C), and the AUC was 0.67 (95% CI: 0.55-0.79).

**Table 3:** Univariable and multivariable analyses of FFRCT non-measurability Data in parentheses are 95% confidence intervals. FFRCT, fractional flow reserve derived from coronary computed tomography angiography; AF, atrial fibrillation

	Univariable analysis		Multivariable analysis	
Variable	Odds ratio	P value	Odds ratio	Odds ratio
Heart rate	1.004 (1.001-1.007]	< 0.01	1.004 (1.001-1.006)	< 0.01
Coronary calcium score	1.0005 (1.0001-1.0009)	0.03	1.0001 (0.9998-1.0004)	0.051
Amount of contrast	1.00 (0.99-1.002)	0.56		
Ejection fraction	0.997 (0.995-0.99)	0.046		
Use of β-blocker	1.03 (0.97-1.09)	0.25		
Use of nitroglycerin	1.05 (0.97-1.14	0.24		
AF at scan	0.93 (0.84-1.03)	0.15		

Predictive features of HR and CCS

**Figure 3:**
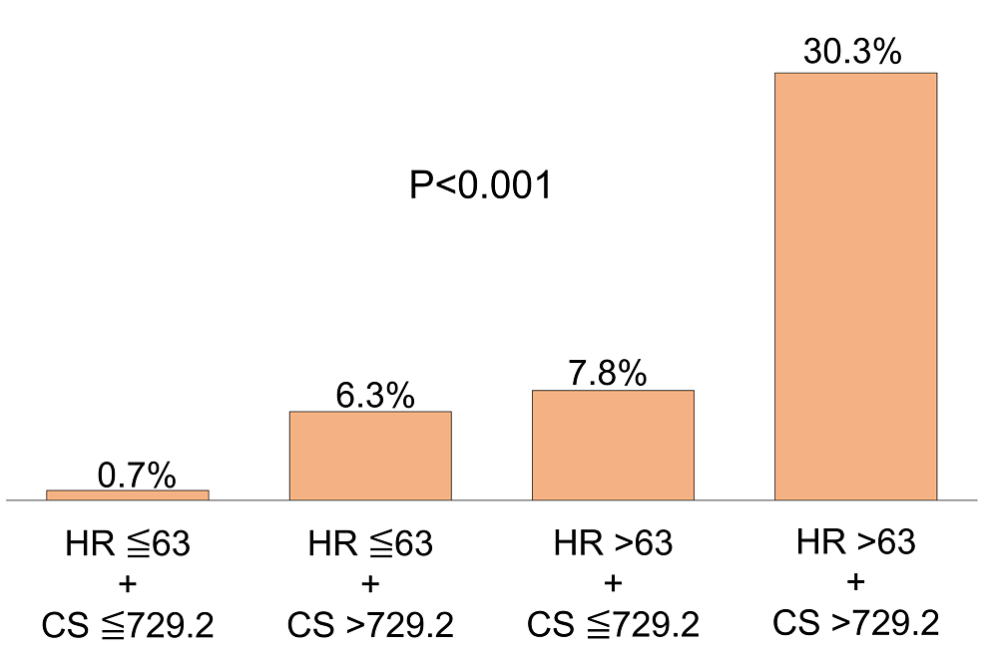
The bar graph of FFRCT non-measurability in each group divided by HR and CCS The FFRCT analysis was most frequently rejected (30.3%) in the group with both a high HR (>63 bpm) and a high CCS (> 729.2), although that was only 0.7% in groups with a low HR (≤ 63 bpm) and a low CCS (≤ 729.2) (p < 0.01). HR, heart rate; CCS, coronary artery calcium score; FFRCT, fractional flow reserve derived from coronary computed tomography angiography

Based on HR and CCS, which were the factors associated with artifact and non-measurability, we classified all cases into four groups and investigated the occupation of non-measurability as follows: low HR (≤ 63) and low CCS (≤ 729.2) (n = 124); low HR (≤ 63) and high CCS (> 729.2) (n = 56); high HR (> 63) and low CCS (≤ 729.2) (n = 87); and high HR (> 63) and high CCS (> 729.2) (n = 40) (Figure 3). Of the four groups, the percentage of non-measurability was the highest in patients with a high HR and high CCS (30.3%), whereas the number of cases with a rejected FFRCT analysis was only one (0.7%) (p < 0.001) in patients with low HR and low CCS. The sensitivity, specificity, positive predictive value, and negative predictive value (NPV) of various characteristics in combination or independently are provided in Table 4. When either the HR was > 63 bpm or the CCS was > 729.2, sensitivity was 95.2% and NPV was 99.3%.

**Table 4:** Predictive features of HR and CCS for FFRCT non-measurability The absence of two features (HR > 63 bpm and CCS > 729.2) demonstrated a high NPV (99.3%) for FFRCT non-measurability. HR, heart rate; CCS, coronary artery calcium score; FFRCT, fractional flow reserve derived from coronary computed tomography angiography; PPV, positive predictive value; NPV, negative predictive value

	Sensitivity (%)	Specificity (%)	PPV (%)	NPV (%)	Accuracy (%)
HR >63 bpm	76.2	67.1	14.5	97.5	67.8
CCS >729.2	66.7	71.3	14.6	96.7	71.0
HR >63 + CCS >729.2	47.6	92.0	30.3	96.0	88.9
HR >63 or CCS >729.2	95.2	46.5	11.6	99.3	49.8

## Discussion

The main findings of our study are that (1) the non-measurability rate due to inadequate image quality was 6.8%, (2) HR was an independent predictor of non-measurability, (3) the optimal HR to complete FFRCT analysis was < 63 bpm, and (4) cases with either an HR of > 63 bpm or a CCS of > 729.2 had extremely high NPV for non-measurability. The points to be emphasized are that HR and CCS are significant predictors associated with non-measurability and notably, considering that we can intervene on HR alone, in cases with severe calcification (CCS of > 729.2), adjustment of HR is particularly recommended because both HR of > 63 bpm and CCS of > 729.2 shows the specificity of 92.0% for non-measurability.

The rate of non-measurability in the current study was lower than that reported in previous studies (8.4-33%) [6,9,14-16]. We considered the major reason as the use of a wide-detector CT scanner. Previous studies have revealed that motion artifacts are closely associated with FFRCT non-measurability [6,9,14], and wide-coverage scanner technology can reduce the occurrence of motion artifacts as they allow whole-heart acquisition for a single beat and can provide relatively accurate images even in cases with a higher HR and arrhythmia [17-19]. In our institution, all CCTA examinations were performed with a 320-detector row CT scanner. In previous studies, the wide-coverage (≥ 16 cm) row scanner was only used in 17-46% of the study population [9,15]. This may be the main reason for the lower rate of non-measurability. Selecting a wide-detector scanner can be one of the methods to obtain optimal FFRCT values, especially in cases with tachycardia or arrhythmias.

Another conceivable reason for the lower non-measurability rate is the thinner CT section. The thickness of the CT section has also been reported as another common predictor for non-measurability in a large cohort study, with thinner sections decreasing the rates of non-measurability [9]. In our institution, all CT data were reconstructed with a 0.25 mm slice thickness, which was thinner than that reported in previous studies (0.63 ± 0.10 mm) [9]. Reconstructing CT images with thinner sections may also reduce image noise [20] and may also be beneficial to increase the rate of completion of FFRCT analysis.

The main reason for the rejection of FFRCT analysis in the present study was motion artifacts, which were the same as those in most previous studies [6,9,15]. Although CT hardware and FFRCT analysis software have improved gradually, HR remains the strongest predictor for non-measurability. To reduce motion artifacts, the Society of Cardiovascular Computed Tomography (SCCT) guidelines recommend HR control under 60 bpm on CT examination [11]. The optimal HR revealed in our analysis (≤ 63 bpm) was very close to the target HR to reduce motion artifacts in CCTA (60 bpm). This may indicate that the FFRCT analysis cannot be accomplished in place of motion artifacts. Therefore, we need to reduce the HR to below 60 bpm during CT scanning. To decrease HR, beta-blocker administration is also recommended [11]. However, in clinical practice, ideal HR control cannot always be accomplished as patients who require CCTA sometimes have aortic stenosis, a low left ventricular ejection fraction, and bronchial asthma, which all are factors that make it difficult for clinicians to use beta-blockers. In fact, the rate of beta-blocker use in this real-world clinical study (45.0%) was lower than in previous studies (72.0-78.0%) [6,21], which were dedicated FFRCT studies with institutions receiving specific FFRCT training and feedback. Thus, in clinical practice, using a wide-detector CT scanner and reconstructing images of better quality may be proper options for obtaining FFRCT values, especially in patients with a higher HR.

In addition, the present study showed that the RCA was the most common non-measurable vessel resulting from motion artifacts. We considered the reason was that the RCA moves in the perpendicular direction to the plane of the CT scan [22].

Apart from motion artifact, blooming artifact was the second cause of non-measurability in our study. However, the number of cases with non-measurability due to blooming artifacts was relatively small. In fact, FFRCT analysis was accomplished in 85.4% of patients with a high CCS (> 729.2), which indicated that FFRCT could be measured even in severely calcified coronary arteries. Basically, in the FFRCT computation processes, luminal dimensions are assessed along the entire length of each vessel using segmentation methods, which can correct calcium blooming [23]. Moreover, it has been demonstrated that FFRCT can provide superior diagnostic performance than CCTA alone in cases with a high CCS [24]. In clinical practice, poor CT images due to massive calcification could make physicians hesitant to undertake FFRCT analysis. However, research has shown that FFRCT can be analyzed completely and provide significant diagnostic performance even in patients with a high CCS.

Finally, we discuss the utility of CCS and HR in predicting the feasibility of FFRCT analysis based on Table 4. For CCS, even at values greater than 729.2, the positive predictive value is only 14.6%, indicating that FFRCT measurement is possible in many cases, as mentioned above. However, if CCS is below 729.2, FFRCT measurement is feasible with a very high likelihood considering a negative predictive value of 96.7%. Additionally, in cases with CCS greater than 729.2, and an HR at CT scan greater than 63, we can expect a very high probability of FFRCT non-measurement considering the specificity of 92.0%. Therefore, it is recommended to keep the HR below 63, particularly in cases of severe calcification. Conversely, as indicated by a sensitivity of 95.2% and a negative predictive value of 99.3%, if the calcification is relatively minor (CCS ≤ 729.2) and HR is controlled within the range of under 63, FFRCT analysis is likely feasible in almost all cases. The same can be applied to HR. FFRCT measurement is possible with an HR greater than 63 bpm, but a lower HR (≤ 63 bpm) increases the probability of successful FFRCT analysis. To note, considering that we can intervene on HR alone, in cases with severe calcification, adjustment of HR is particularly recommended.

Limitations

This study had several limitations. First, the present study was a retrospective analysis of data collected from a single center, representing a small cohort of FFRCT cases in Japan. Thus, selection biases are inevitable, and our study was underpowered to make strong statements. Validation in large multicenter prospective studies is required. The second limitation was referral bias. The decision to proceed to FFRCT analysis was made by each physician interpreting the CCTA, and referral bias was likely present. Third, we did not investigate all factors that had a significant association with non-measurability in previous studies, including temporal resolution, aorta contrast opacification, reconstructed field of view, and atheroma volume. Since these can be factors associated with CCTA image quality and confounders for non-measurability, future studies will be required to collect these variables.

## Conclusions

The rate of non-measurability of FFRCT was 6.8% in our hospital. HR and CCS were important factors in acquiring FFRCT values and in cases with both HR and CCS below a specified threshold, the likelihood of ruling out non-measurability could be significantly high. Particularly in cases with severe calcification (CCS > 729.2), adjustment of HR to under 63 bpm is highly recommended considering that the specificity of both HR > 63 bpm and CCS > 729.2 for non-measurablity was 92%. Furthermore, using a wide-detector CT scanner and reconstructing images of better quality may be proper options for obtaining FFRCT values, especially in patients with a higher HR. While technological advancements in CCTA machines and FFRCT analysis software are expected, further investigation in a larger-scale cohort will help reduce the non-measurability rate more by elucidating the valuable predictive factors that we can adjust.
